# Mapping the transcriptional evolution of human metastatic breast cancer

**DOI:** 10.1172/JCI183971

**Published:** 2024-09-03

**Authors:** Melissa Q. Reeves

**Affiliations:** 1 Huntsman Cancer Institute, and; 2 Department of Pathology, University of Utah, Salt Lake City, Utah, USA.

## Abstract

Many aspects of breast cancer metastasis remain poorly understood, despite its clinical importance. In this issue of the *JCI*, Winkler et al. have applied an elegant patient-derived xenograft (PDX) model to map the transcriptomes of single cells in matched primary tumors and lung metastases across 13 breast cancer PDX models. They identified distinct transcriptional changes associated with metastatic evolution in lowly and highly metastatic primary tumors. Furthermore, by classifying the “epithelial-mesenchymal plasticity” (EMP) state of single cells, they revealed that considerable EMP heterogeneity exists among primary and metastatic human breast cancer cells. However, the EMP profile of a tumor does not change substantially upon metastasis. These findings give an unprecedentedly detailed view into the transcriptional heterogeneity and evolution of metastatic human breast cancer.

## Epithelial and mesenchymal programs in metastasizing tumor cells

Metastasis is a complex biological process that has been studied for over two centuries (1–4). Yet, it remains a major cause of death for patients with cancer, and many aspects of its mechanistic drivers remain elusive (2, 4). Metastasis involves several steps, including local invasion, survival in circulation, and outgrowth at a distant site, and during this process, metastasizing tumor cells must evade immune surveillance (1, 2). It has been appreciated for over a decade now that tumor cells that lose epithelial properties and gain mesenchymal ones — referred to as epithelial-mesenchymal transition (EMT) — have an advantage in metastasizing (1–3). However, many macrometastases are observed to have retained or reacquired epithelial properties, adding nuance to the widely accepted concept that EMT is associated with metastasis. EMT is now recognized not to be a sudden binary switch from one state to another,but rather a continuum of epithelial-like, intermediate (or hybrid), and mesenchymal-like states that tumor cells can exist along (2, 5–8). Recent evidence suggests that cells existing in a hybrid EMT state may in fact be the most metastatically aggressive (7, 8). Additionally, clusters of tumor cells that travel together — referred to as circulating tumor cell (CTC) clusters — have also been identified in patients with breast cancer and in mouse models (9–12), and exhibit greater metastatic efficiency than do single CTCs (9, 11, 12). Tumor cells participating in CTC clusters maintain cell-cell contacts and exhibit differences in transcriptional and methylation patterns, including increased expression of epithelial genes (12, 13) and more open chromatin around stemness-associated genes (10), compared with solitary CTCs. Thus, the journey to metastasis is far more nuanced than a one-way trip toward an increased mesenchymal program.

In this issue of the *JCI*, Winkler and colleagues have undertaken deep single-cell transcriptomic characterization of 13 highly and lowly metastatic human breast cancer patient–derived xenograft (PDX) models (14). The transcriptional landscape of tumor cells was compared between PDX-derived primary tumors and matched, spontaneously arising lung metastases. This elegant approach offers a unique view into the transcriptional evolution of metastatic human breast cancer. In patients with breast cancer, it is common for primary tumors and metastases to be separated by years, or even a decade or more (4, 15). Not only does this make it logistically challenging to collect matched primary and metastatic tumor samples, but it means latent or dormant metastatic tumor cells can be subject to a whole range of influences — from adjuvant treatments to lifestyle changes or environmental exposures the patient experiences — in the years between the presentation of the primary tumor and the metastasis. It is virtually impossible to disentangle how these factors impinge on the genetic and transcriptional programs of tumor cells that eventually grow out as metastasis (15). In their clean PDX system, Winkler and colleagues were uniquely able to directly compare the transcriptional landscape of matched primary and metastatic human breast cancer cells.

Using an epithelial-mesenchymal plasticity (EMP) score assigned to each primary-derived and metastasis-derived single cell, Winkler and colleagues classified each tumor cell in their PDX models as “epithelial-like,” “EMP intermediate,” or “mesenchymal-like”(14). All 13 tumor models contained cells belonging to at least two of these three categories, demonstrating that intratumoral heterogeneity of EMT and EMP states is a strong feature of human tumors, in line with predictions made with murine models (7, 8). Furthermore, a gene signature associated specifically with “EMP intermediate” cells predicted worse recurrence-free survival in patients with basal subtype or HER2-like breast cancer (14). While this signature lacks prognostic association in luminal subtypes, it is perhaps worth noting that 10 of the 13 models utilized in Winkler and colleagues’ study were of the basal subtype, and their signature may have been particularly well suited to identify “intermediate EMP” cells in the basal subtype of breast cancer.

However, despite this association between the “intermediate EMP” signature and poorer outcomes for patients, the abundance of “intermediate EMP” cells was surprisingly not different between highly and lowly metastatic PDX models (14). On the other hand, within each breast cancer subtype, there was a notable shift toward fewer epithelial-like and more mesenchymal-like cells in the primary tumors from highly metastatic models. This raises a question about what features of EMT and EMP are truly most relevant to predicting metastasis: Is it the abundance of intermediate cells, the abundance of mesenchymal cells, or something else? While future work will likely shed more light on the answers to this question, a limitation of single-cell RNA-Seq (scRNA-Seq) is that it can only capture the distribution of single cells across states at a single moment in time. Single-cell transcriptome sequencing gives little visibility of the flux between these states. Given that the process of metastasis requires cells to rapidly adapt to multiple environments and sources of cellular stress, it is possible that the speed at which a tumor cell can transition between EMT and EMP states is equally important to its metastatic potential. Technologies that can assess this transcriptional flux at scale will be important to future advances in the field.

## Transcriptional evolution trajectories differ with metastatic potential

The unique dataset generated by Winkler and colleagues enables an inquiry into transcriptional evolution that takes place between primary tumor and metastasis, in a model system in which other environmental factors can be completely controlled. Surprisingly, the distribution of EMP states found in metastases largely matched that of their primary tumor of origin. This result means that the transcriptional evolution during metastasis did not include a shift in EMP states. Nonetheless, each PDX model clearly underwent a transcriptional evolution that was unique to that model but that was consistent across multiple mice bearing the same PDX (14). While this trend did not correspond with genetic evolution, it nonetheless suggests that the evolutionary trajectory of metastasis is precoded in some way in each PDX. Winkler and colleagues looked for commonalities between the trajectories of tumors with similar metastatic potential. Genes associated with motility were commonly upregulated in the metastases of tumors with low metastatic potential, whereas genes associated with the stress response were commonly upregulated in metastasis of cells with highly metastatic potential (14). This finding raises the possibility that tumors have different requirements to reach the metastatic state, depending on their starting transcriptional state and phenotype. It is readily inferable that tumor cells in lowly metastatic tumors may have a greater need to acquire increased cell motility, while tumor cells in highly metastatic PDXs — with more cells already in a mesenchymal-like state — have already acquired this feature. However, less clear is the reason tumor cells originating in highly metastatic tumors are unique in their upregulation of stress response genes upon metastasis. It is possible that the upregulation of these genes was simply dwarfed in lowly metastatic tumors by a more critical upregulation of motility genes. Alternatively, metastatic tumor cells originating from highly metastatic tumors might experience more aggressive competition with one another to seed and expand at the metastatic site, which could shape the evolutionary trajectory of the cells that succeed in outcompeting their neighbors.

## Immune surveillance in metastasis and EMT

Of course, a footnote to all studies in immune-compromised mice relates to their lack of an immune system. Successful immune evasion is a critical component of the full metastatic cascade (2). NK cells, which were lacking in the NSG mice used in this study, are particularly known for having an important role in immune surveillance of metastatic tumor cells (13, 16). Notably, NK cells are capable of preferentially eliminating single CTCs over CTC clusters (13). Thus, it is possible that removing the pressure of NK cell surveillance might lead to an overrepresentation of metastases from single CTCs, relative to those arising from CTC clusters, and influence the transcriptome of metastases that successfully grow out. Additionally, an intriguing recent study carried out by the Blanpain laboratory used anti-Csf1r and anti-Ccl2 antibodies to ablate macrophages; their data showed that tumor cells in macrophage-deficient tumors were more likely to adopt an epithelial or early hybrid EMT state and less likely to adopt a mesenchymal state (7). NSG mice do have macrophages, and so while this point may not have directly related to Winkler et al., it highlights how much remains to be learned about the interplay between immune cells, metastasis, and EMT.

## Conclusions

**Figure 1:**
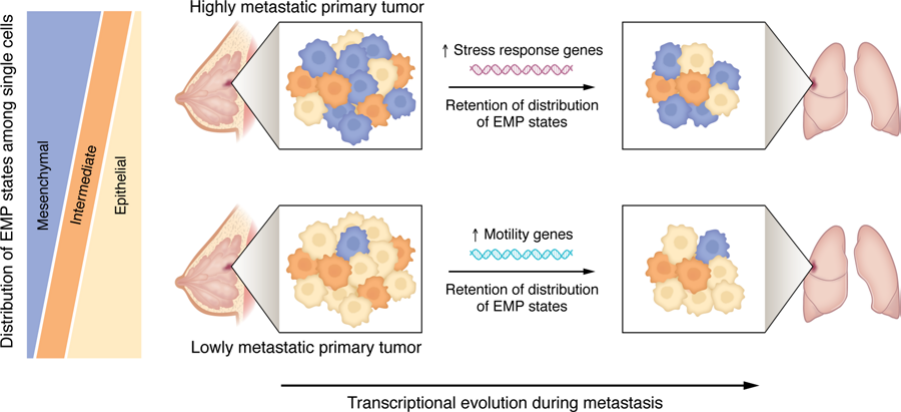
Transcriptional heterogeneity and evolution of metastatic breast cancer. Human breast cancer cells exhibit substantial intratumoral heterogeneity of EMP states. Within a given breast cancer subtype, more highly metastatic primary tumors are more likely to contain a larger proportion of mesenchymal-like cells compared with lowly metastatic tumors. Upon metastasis to the lung, the EMP profile of the primary tumor is retained. Transcriptional evolution during metastasis, instead of involving changes in the EMP profile, is characterized by upregulation of stress response genes in highly metastatic primary tumors, or of motility genes in lowly metastatic primary tumors.

The study by Winkler and colleagues sheds valuable light on the transcriptional evolution of human metastatic breast cancer. The authors define distinct transcriptional trajectories — one relying on increases in cell motility, and the other relying on increases in the stress response — taken by lowly and highly metastatic primary tumors, respectively, to arrive at the metastatic stage. Furthermore, despite the importance of EMT and EMP in metastasis and the ability of an intermediate EMP signature to predict worse outcomes, the distribution of EMP states does not change between a primary tumor and its matched metastasis (Figure 1) (14). In future work, an interesting open question involves how these patterns may differ between short and long intervals of metastatic dormancy. By necessity, Winkler et al. (14) focused on metastases that arose within months of the primary tumor occurrence. Future studies may be able to test how the paradigms put forth here translate to, or need to be modified for, patients whose breast cancer recurs after a decade or more, as well as to metastases that spread to other anatomical sites.

## Version 1 09/03/2024

Electronic publication

## References

[1] Valastyan S, Weinberg RA (2011). Tumor metastasis: molecular insights and evolving paradigms. Cell.

[2] Lambert AW (2017). Emerging biological principles of metastasis. Cell.

[3] Talmadge JE, Fidler IJ (2010). AACR centennial series: the biology of cancer metastasis: historical perspective. Cancer Res.

[4] Riggio AI (2021). The lingering mysteries of metastatic recurrence in breast cancer. Br J Cancer.

[5] Sampson VB (2014). Wilms’ tumor protein induces an epithelial-mesenchymal hybrid differentiation state in clear cell renal cell carcinoma. PLoS One.

[6] Schliekelman MJ (2015). Molecular portraits of epithelial, mesenchymal, and hybrid States in lung adenocarcinoma and their relevance to survival. Cancer Res.

[7] Pastushenko I (2018). Identification of the tumour transition states occurring during EMT. Nature.

[8] Simeonov KP (2021). Single-cell lineage tracing of metastatic cancer reveals selection of hybrid EMT states. Cancer Cell.

[9] Aceto N (2014). Circulating tumor cell clusters are oligoclonal precursors of breast cancer metastasis. Cell.

[10] Gkountela S (2019). Circulating tumor cell clustering shapes DNA methylation to enable metastasis seeding. Cell.

[11] Maddipati R, Stanger BZ (2015). Pancreatic cancer metastases harbor evidence of polyclonality. Cancer Discov.

[12] Cheung KJ (2016). Polyclonal breast cancer metastases arise from collective dissemination of keratin 14-expressing tumor cell clusters. Proc Natl Acad Sci U S A.

[13] Lo HC (2020). Resistance to natural killer cell immunosurveillance confers a selective advantage to polyclonal metastasis. Nat Cancer.

[14] Winkler et al (2024). Single-cell analysis of breast cancer metastasis reveals epithelial-mesenchymal plasticity signatures associated with poor outcomes. J Clin Invest.

[15] Savas P (2016). The subclonal architecture of metastatic breast cancer: results from a prospective community-based rapid autopsy program “CASCADE”. PLoS Med.

[16] Malladi S (2016). Metastatic latency and immune evasion through autocrine inhibition of WNT. Cell.

